# Electrochemical and Thermodynamic Properties of Ln(III) (Ln = Eu, Sm, Dy, Nd) in 1-Butyl-3-Methylimidazolium Bromide Ionic Liquid

**DOI:** 10.1371/journal.pone.0095832

**Published:** 2014-04-21

**Authors:** Xiao Yang, Ling He, Song Qin, Guo-Hong Tao, Ming Huang, Yi Lv

**Affiliations:** 1 College of Chemistry, Sichuan University, Chengdu, Sichuan, China; 2 Institute of Chemical Materials, China Academy of Engineering Physics, Mianyang, Sichuan, China; Queen’s University Belfast, United Kingdom

## Abstract

The electrochemical behavior and thermodynamic properties of Ln(III) (Ln = Eu, Sm, Dy, Nd) were studied in 1-butyl-3-methylimidazolium bromide ionic liquid (BmimBr) at a glassy carbon (GC) electrode in the range of 293–338 K. The electrode reaction of Eu(III) was found to be quasi-reversible by the cyclic voltammetry, the reactions of the other three lanthanide ions were regarded as irreversible systems. An increase of the current intensity was obtained with the temperature increase. At 293 K, the cathodic peak potentials of −0.893 V (Eu(III)), −0.596 V (Sm(III)), −0.637 V (Dy(III)) and −0.641 V (Nd(III)) were found, respectively, to be assigned to the reduction of Ln(III) to Ln(II). The diffusion coefficients (*D*_o_), the transfer coefficients (*α*) of Ln(III) (Ln = Eu, Sm, Dy, Nd) and the charge transfer rate constants (*k*_s_) of Eu(III) were estimated. The apparent standard potential (*E* ^0^*) and the thermodynamic properties of the reduction of Eu(III) to Eu(II) were also investigated.

## Introduction

Ionic liquids (ILs) have attracted much attention in recent years as interesting soft materials including solvents [1]–[7], catalysts [8]–[15], lubricants [16], electrolytes [17]–[21], extractants [22]–[23], magnetic fluids [24]–[26], optical fluids [27]–[28], and propellants [29]–[33]. Compared with conventional molecular solvents, ILs have unique physical properties such as high thermal stability, large liquidus range, negligible vapor pressure, and wide electrochemical window [34]–[36]. ILs are typical ionic compounds, and therefore normally have high electrical conductivities and good charge transport properties. Combined with their distinctive solvation ability to a wide variety of inorganic, organic, and organometallic species, ILs have inherent advantages to be used for various electrochemical applications [37]–[39].

Lanthanide elements present fascinating and intricate properties in view of singular photophysical/optical, catalytic, and magnetic properties [40]. Along with the increased demand of rare earths, there is a growing interest in high pure lanthanides. Selective separation of lanthanides is necessary for their applications [41]. Furthermore, in the nuclear fuel cycle, the technology of the selective separation of lanthanides is of importance for maximum utilization of the expensive nuclear fuel resource. ILs have shown potential as a solvent in nuclear fuel reprocessing technology [42]. The separation coefficients of lanthanides are related to formal standard potentials, transfer and diffusion coefficients, and standard rate constants of charge transfer [43]. These electrochemical properties of lanthanides will be very important for the selective separation of lanthanides in ILs.

The electrochemical behavior of Ln(III) in room temperature ILs composed of bis(trifluoromethylsulfonyl)imide anion (NTf_2_^−^) was studied. Yamagata et al. studied the electrochemical behavior of Eu(III), Sm(III), Yb(III) in 1-butyl-1-methylpyrrolidinium bis(trifluoromethylsulfonyl)imide (BMPyNTf_2_) and 1-ethyl-3-methylimidazolium bis(trifluoromethylsulfonyl)imide (EMINTf_2_) ILs, which exhibit large electrochemical windows [44]. The cyclic voltammograms of these lanthanide ions consisted of quasi-reversible waves that were attributed to the reduction of these trivalent lanthanides to their respective divalent states and the oxidation of divalents to their respective trivalents. The diffusion coefficients of these trivalent lanthanides in these ILs were determined to be ∼10^−8 ^cm^2^·s^−1^. Rao et al. reported the electrochemical behavior of Eu(III) in BMPyNTf_2_ IL [45]. Cyclic voltammogram of Eu(III) consisted of a quasi-reversible cathodic wave at −0.45 V (vs. Fc/Fc^+^, 373 K), which could be attributed to the reduction of Eu(III) to Eu(II) and an irreversible wave at −2.79 V (vs. Fc/Fc^+^) assigned to the reduction of Eu(II) to Eu(0). The diffusion coefficient of Eu(III) in BMPyNTf_2_ was determined to be about ∼10^−7 ^cm^2^·s^−1^, and the charge transfer rate constant (*k*_s_) was ∼10^−5 ^cm·s^−1^ by cyclic voltammetry. Matsumiya et al. got the diffusion coefficients of Eu(III) and Sm(III) in NTf_2_^−^ ILs to be ∼10^−12 ^cm^2^·s^−1^ [46]. Bhatt et al. studied the electrochemical behaviors of La(III), Sm(III) and Eu(III) in tetraalkylammonium bis(trifluoromethylsulfonyl)imide ILs [47]–[49]. The above investigations indicate that ILs are proposed as efficient candidates for high-temperature molten salts in non-aqueous reprocessing.

The relative expensive cost of ILs is a key factor limiting their applications. ILs based on NTf_2_^−^ are high-cost, though they exhibit good fluidity. Some low-cost alternatives with good physicochemical properties are of interest. 1-Butyl-3-methylimidazolium bromide (BmimBr), is a classical ionic liquid, with a melting point of 76°C [50]. However, BmimBr usually exhibits supercool status as a liquid at room temperature for a long time. This ionic liquid can be obtained in a big scale with low cost via a simple synthesis route skipping the tough purification of water. Such features suggest BmimBr can be regarded as a potential candidate for industry application. Water is an inevitable impurity in nearly all ILs, even for the hydrophobic NTf_2_^−^ and PF_6_^−^ ILs. Small amounts of water residual in BmimBr may lead to an increase of its electrical conductivity, and a decrease of its viscosity and melting point. BmimBr obtained by simple methods is a realistic consideration in its potential applications. Herein, the electrochemical behaviors of four trivalent lanthanides, Eu(III), Sm(III), Dy(III) and Nd(III), were investigated in BmimBr by cyclic voltammetry. Their diffusion coefficients and transfer coefficients were estimated. The thermodynamic properties of Eu(III) including charge transfer rate constant, formal potential, and Gibbs energy were also studied.

## Results and Discussion

### 1. Cyclic Voltammetry

**Figure 1:**
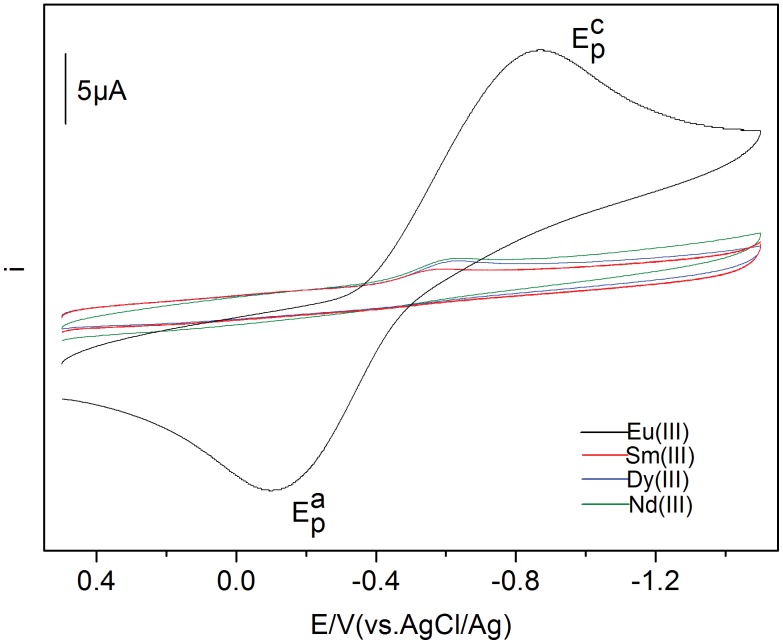
Cyclic voltammograms of Eu(III), Sm(III), Dy(III) and Nd(III) measured in BmimBr at 293 K.

The cyclic voltammograms of Ln(III) (Ln = Eu, Sm, Dy, Nd, 50 mmol·L^−1^) in BmimBr at 293 K are shown in Figure 1. For Eu(III), a cathodic peak and an anodic peak potentials were observed around −0.893 V and −0.121 V, respectively. The cathodic and anodic peaks were attributed to the reduction and oxidation of Eu(III) separately. No deposition of europium metal was observed after the potentiostatic reduction. Therefore, this result shows that the reduced product was the divalent europium complex, Eu(II). The redox reaction of the Eu(III)/Eu(II) in BmimBr is a quasi-reversible. The cyclic voltammograms of Sm(III), Dy(III) and Nd(III) gave irreversible waves. Their cathodic peak potentials were around −0.596 V (Sm(III)), −0.637 V (Dy(III)) and −0.641 V (Nd(III)), respectively, which are higher than that of Eu(III). The difference in the curves of Sm(III), Dy(III) and Nd(III) is perhaps due to the instability of Sm(II), Dy(II) and Nd(II) to Eu(II). Relative to Eu(II), the other three Sm(II), Dy(II) and Nd(II) are easily oxidized to their respective trivalent states in BmimBr, leading to no anodic peak observed in the cyclic voltammograms. This property also affected the cathodic peaks of Sm(III), Dy(III) and Nd(III).

**Figure 2:**
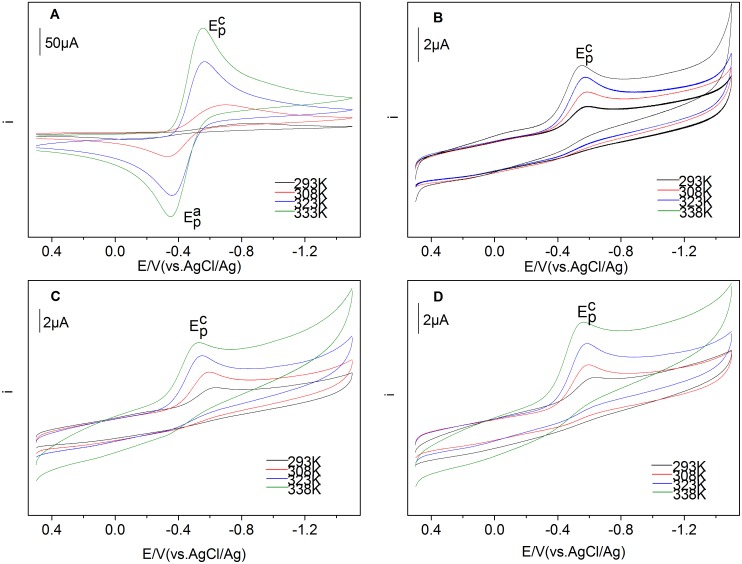
Cyclic voltammograms of Eu(III) (A), Sm(III) (B), Dy(III) (C) and Nd(III) (D) measured in BmimBr at different temperatures.

Figure 2 shows the cyclic voltammograms of 50 mmol·L^−1^ Ln(III) (Ln = Eu, Sm, Dy, Nd) at various temperatures. Temperatures were well controlled and selected as 293 K, 308 K, 323 K and 338 K, respectively. For Eu(III) in BmimBr, the current intensities increased along with the rise of temperature. A similar tendency was found for the other three Ln(III): Sm(III), Dy(III) and Nd(III). This feature is associated with the mass transition caused by the viscosity of BmimBr, which depends on temperature closely [36]. Thus, the transport properties of ILs, including conductivity, diffusion coefficient, and charge transfer rate are also temperature-dependent for the variation of viscosity. For BmimBr ionic liquid, its viscosity decreased and the conductivity increased at higher temperature, which would facilitate the diffusion of trivalent lanthanide ions. The diffusion rate became greater as the temperature increased.

**Figure 3:**
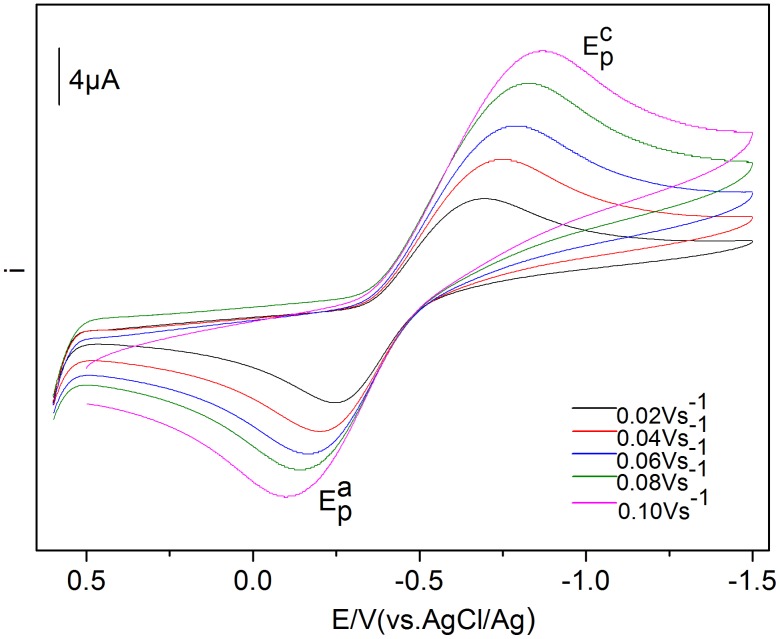
Cyclic voltammograms of Eu(III) measured in BmimBr with different scan rates.

The cyclic voltammograms of 50 mmol·L^−1^ Eu(III) in BmimBr at various scan rates are described in Figure 3. Both current intensity and peak potential were changed, along with the change of scan rate. The cathodic and anodic peak potentials were shifted to cathode and anode, respectively, with the increase of scan rate.

**Figure 4:**
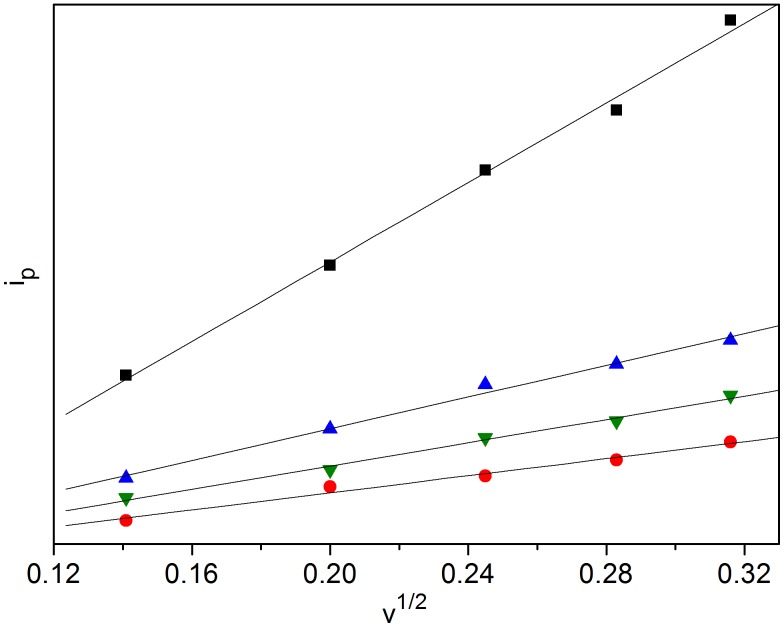
Plots of cathodic peak current intensity (*i*_p_) against square-root of the potential scan rate (*ν* ^1/2^); ▪, Eu(III); •, Sm(III); ▴, Dy(III); ▾, Nd(III).

The plots of cathodic peak current intensity (*i*_p_) against the square-root of the potential scan rate (*ν* ^1/2^), are shown in Figure 4. For Eu(III), a positive correlation of the current intensity with the scan rate was determined. This result indicates that the electrode reaction kinetics is controlled by the mass transport under semi-infinitive linear diffusion conditions. Good linear relations were also observed for Sm(III), Dy(III) and Nd(III) in BmimBr.

### 2. Diffusion Coefficients (*D*_o_), Transfer Coefficients (*α*) and the Energy of Activation (*E*_a_) of Eu(III), Sm(III), Dy(III) and Nd(III) in BmimBr

From a series of electrochemical analyses, the diffusion coefficients of Ln(III) in BmimBr were estimated. For an irreversible or quasi-reversible system, the relation of the cathodic peak current and the diffusion coefficient (*D*_o_) can be predicted as equation (1) [51], [52]:
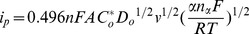
(1)where *A* is the electrode area in cm^2^ (0.1256), *C*_o_ * is the Ln(III) concentration in mmol·L^−1^ (∼50 mmol·L^−1^), *D*_o_ is the diffusion coefficient in cm^2^·s^−1^, *ν* is the potential scan rate in V·s^−1^, *F* is the Faraday constant, *α* is the charge transfer coefficient, *n* is the number of transferred electrons, *n*_α_ is the number of electrons transferred in the rate determining step, and *T* is the absolute temperature in K. The value of *αn*_α_ can be estimate as equation (2) [45]:
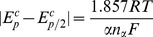
(2)where *E*_p_^c^ is the cathodic potential, *E*_p/2_^c^ is the half wave potential, and |*E*_p_^c^−*E*_p/2_^c^| is the absolute value of the difference between *E*_p_^c^ and *E*_p/2_^c^. These data of Ln(III) recorded at different temperatures are summarized in Table 1. For Ln(III) (Ln = Eu, Sm, Dy, Nd), the value of *n*_α_ is 1.

**Table 1:** Peak potentials *E*_p_^c^, *E*_p/2_^c^ and |*E*_p_^c^−*E*_p/2_^c^| of Eu(III), Sm(III), Dy(III) and Nd(III) in BmimBr at different temperatures.

Metal ion	*T*/K	*E*_p_^c^/V	*E*_p/2_^c^/V	|*E*_p_^c^−*E*_p/2_^c^|/V
Eu(III)	293	−0.893	−0.582	0.311
	308	−0.639	−0.466	0.173
	323	−0.565	−0.460	0.105
	338	−0.557	−0.455	0.102
Sm(III)	293	−0.596	−0.369	0.227
	308	−0.587	−0.421	0.166
	323	−0.579	−0.430	0.149
	338	−0.553	−0.406	0.147
Dy(III)	293	−0.637	−0.412	0.225
	308	−0.596	−0.441	0.155
	323	−0.551	−0.409	0.142
	338	−0.545	−0.406	0.139
Nd(III)	293	−0.641	−0.425	0.216
	308	−0.602	−0.449	0.153
	323	−0.591	−0.442	0.149
	338	−0.562	−0.413	0.149

According to equations (1) and (2), the diffusion coefficients of Ln(III) in BmimBr can be estimated. The values of *D*_o_ and *α* are given in Table 2. The diffusion coefficients can be regarded as a function of *T*. An increase of the diffusion coefficients and the charge transfer coefficients with temperature is observed. The diffusion coefficient of Eu(III) is about 10^−8 ^cm^2^·s^−1^ at 293 K, and as high as ∼10^−7 ^cm^2^·s^−1^ at higher temperature. Low viscosity of ILs at high temperature results in more efficient mass transport. Similar trends are also found for Sm(III), Dy(III) and Nd(III) in BmimBr, however, no increase in their magnitude of diffusion coefficient was found. The magnitude of diffusion coefficients of Sm(III), Dy(III) and Nd(III) is around 10^−10 ^cm^2^·s^−1^, which is 10^2^∼10^3^ times smaller than that of Eu(III) in BmimBr. This fact indicates that the electrostatic interaction around Eu(III) in BmimBr may be weaker than those of Sm(III), Dy(III) and Nd(III). At 338 K, the diffusion coefficient of Eu(III) increased more than those of the other three Ln(III).

**Table 2:** Diffusion coefficients (*D*_o_), transfer coefficients (*α*) and energy of activation (*E*_a_) of Eu(III), Sm(III), Dy(III) and Nd(III) in BmimBr at different temperatures.

Metal ion	*T*/K	*D*_o_×10^10^/cm^2^·s^−1^	*E*_a_/kJ·mol^−1^	*α*
Eu(III)	293	96.86	59.09	0.151
	308	271.2		0.285
	323	1171		0.492
	338	2285		0.530
Sm(III)	293	0.7561	18.94	0.206
	308	1.114		0.296
	323	1.558		0.347
	338	2.110		0.368
Dy(III)	293	1.092	27.22	0.208
	308	1.882		0.318
	323	2.987		0.364
	338	4.813		0.389
Nd(III)	293	1.054	20.39	0.217
	308	1.416		0.322
	323	2.203		0.347
	338	3.108		0.363

**Figure 5:**
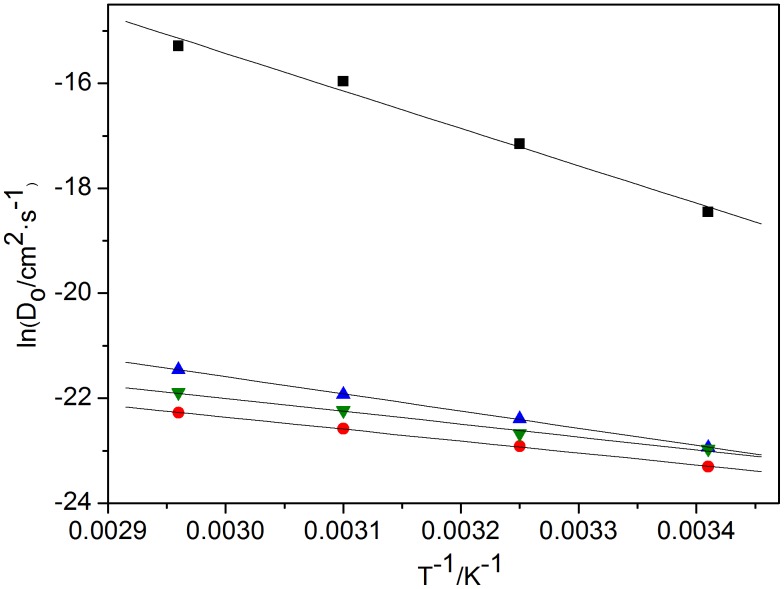
Plots of ln*D*_o_ against *T* ^−1^, ▪, Eu(III); •, Sm(III); ▴, Dy(III); ▾, Nd(III) measured in BmimBr at GC electrode.

From the slope of ln*D*_o_ against 1/*T*, the energy of activation (*E*_a_) of the reduction of Ln(III) to Ln(II) can be determined. (Figure 5, Table 2) The reduction of Eu(III) to Eu(II) exhibits the highest value of *E*_a_ of 59.09 kJ·mol^−1^. While, the values of other three Ln(III) are found around 20 kJ·mol^−1^ (Sm(III), 18.94 kJ·mol^−1^; Dy(III), 27.22 kJ·mol^−1^; and Nd(III), 20.39 kJ·mol^−1^).

### 3. Charge Transfer Rate Constants (*k*_s_) of Eu(III) in BmimBr

Diffusion and charge transfer kinetics are the major factors that affect the reduction of Ln(III) to Ln(II) in BmimBr. The charge transfer rate constant (*k*_s_, cm·s^−1^), associated with both diffusion coefficient and transfer coefficient, can be described as equation (3) [53]:
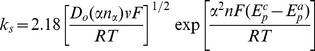
(3)


The charge transfer rate constants (*k*_s_), the cathodic and anodic peak potentials (*E*_p_^c^ and *E*_p_^a^) of Eu(III) in BmimBr at different temperatures are given in Table 3. The magnitude of charge transfer rate constants are found to be ∼10^−5 ^cm·s^−1^. Such data increases when the temperature increases. The lower viscosity of BmimBr at higher temperature may advantage electron transfer at electrode-electrolyte interphase. Thus, an increase of *k*_s_ for Eu(III) in BmimBr can be found at higher temperature. According to the value of *k*_s_, the electrode reaction can be summarized to be reversible (*k*_s_≥0.3 *ν* ^1/2 ^cm·s^−1^), quasi-reversible (0.3 *ν* ^1/2^≥ *k*_s_≥2×10^−5^ *ν* ^1/2 ^cm·s^−1^), and irreversible (*k*_s_≤2×10^−5^ *ν* ^1/2 ^cm·s^−1^). A quasi-reversible electrode reaction of Eu(III) to Eu(II) is confirmed by the values of *k*_s_.

**Table 3:** Rate constants (*k*_s_), peak potentials (*E*_p_^c^ and *E*_p_^a^) of Eu(III) in BmimBr at different temperatures.

Metal ion	*T*/K	*E*_p_^c^ */*V	*E*_p_^a^/V	*k*_s_×10^5^/cm·s^−1^
Eu(III)	293	−0.893	−0.121	7.422
	308	−0.639	−0.320	14.37
	323	−0.565	−0.356	16.23
	338	−0.557	−0.352	19.07

### 4. Determination of Gibbs Energy Change of Eu(III) in BmimBr

**Figure 6:**
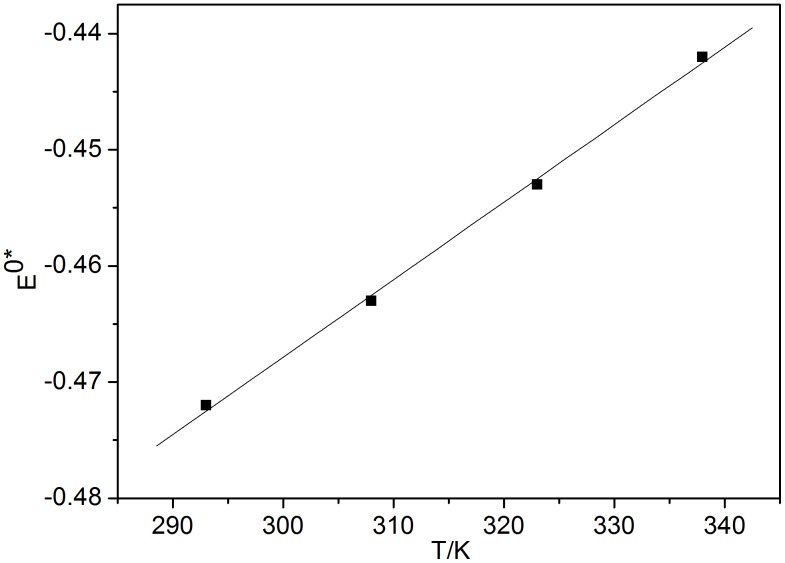
Plots of *E* ^0^*_Eu(III)/Eu(II)_ against *T*.



Gibbs energy, Δ*G*, is of central importance to reaction. The reduction of Eu(III) to Eu(II) in BmimBr is simply described as:
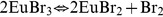
(4)For a dilute solution system, its activity coefficient is negligible. Assuming Eu(III) in BmimBr forms a dilute solution, thus, the standard Gibbs energy of the reaction EuBr_2_+1/2Br_2_ → EuBr_3_ can be identified by the expression (5).
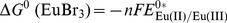
(5)where *E* ^0*^_Eu(II)/Eu(III)_ is the apparent standard potential of oxidation of Eu(II) to Eu(III). The apparent standard potential, *E* ^0*^_Eu(III)/Eu(II)_, is associated with its cathodic and anodic peak potentials. The relation between *E* ^0*^_Eu(III)/Eu(II)_ and *E*_p_^c^ (and *E*_p_^a^) is given as:



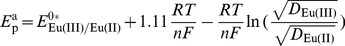
(6)

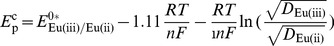
(7)Because the reduction of Eu(III) to Eu(II) involves a single electron transfer, *n* is equal to 1, *E* ^0^*_Eu(III)/Eu(II)_ can be expressed as equation (8), as a function of temperature.
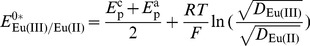
(8)From linear regression of the experimental data, (Figure 6) the equation for the apparent standard potential E0*Eu(III)/Eu(II) (equation (9)) has the form:

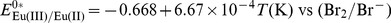
(9)This expression, which shows that a change in *E* ^0^*_Eu(III)/Eu(II)_ is proportional to a change in *T*, suggests that *E* ^0^*_Eu(III)/Eu(II)_ can be regarded as a function of *T*. A linear correlation of *E* ^0^*_Eu(III)/Eu(II)_ with temperature is found.

Based on equations (5) and (9), the standard Gibbs energy expression is
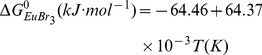
(10)


The standard Gibbs energy is found to be a linear function of temperature. The standard Gibbs energy of reaction is expressed, combined with standard entropy and enthalpy of reaction: Δ*G* = Δ*H*−*T*Δ*S*. The expression of equation (10) shows that the standard entropy (Δ*S* ^0^_EuBr3_) of the reaction EuBr_2_+1/2Br_2_→EuBr_3_ is negative. The result indicates that a decrease in the entropy of the reaction occurs, along with the formation of less disordered EuBr_3_ from higher disordered substrates.

## Materials and Methods

### 1. Chemicals

All chemicals of analytical grade, 1-methylimidazole, 1-bromobutane, Eu_2_O_3_, Sm_2_O_3_, Dy_2_O_3_ and Nd_2_O_3_ were obtained commercially. Infrared spectra (IR) were recorded by KBr plates on a Nicolet NEXUS 670 FT-IR spectrometer. ^1^H and ^13^C NMR spectra were recorded on a Bruker 400 MHz nuclear magnetic resonance spectrometer with CDCl_3_ as locking solvent unless otherwise stated. ^1^H and^ 13^C chemical shifts were reported in ppm relative to TMS. Water content was determined by coulometric Karl-Fischer titration.

Preparation of LnBr_3_ (Ln = Eu, Sm, Dy, Nd). Ln_2_O_3_ (0.25 mmol) was dispersed in trace water. A slightly excess of hydrobromic acid was added into the solution. The resulting mixture was stirred and heated to remove the excess hydrobromic acid. The crude product was dried in vacuum for 12 h at 100°C to yield the desired LnBr_3_·nH_2_O.

Synthesis of 1-butyl-3-methylimidazolium bromide (BmimBr). 1-Methylimidazole (32.84 g, 400 mmol) was dissolved in toluene (50 mL). 1-Bromobutane (57.55 g, 420 mmol) was added dropwise into the resultant solution with vigorous stirring at room temperature. The resulting mixture was stirred for 24 h at room temperature. The toluene layer was removed, and the residue was dried in vacuum for 1 h at 80°C to produce a colorless transparent liquid. (61.68 g, 94%) Water content 1.35 wt%. IR (KBr, cm^−1^): 3125 (s), 3081 (vs), 2956 (vs), 2868 (vs), 1566 (vs), 1461 (vs), 1168 (vs), 755 (m). ^1^H-NMR (CDCl_3_, *δ*/ppm): 9.98 (s, 1H), 7.51 (s, 1H), 7.38 (s, 1H), 4.07 (t, 2H, *J* = 7.2 Hz), 3.84 (s, 3H), 1.60 (m, 2H), 1.07 (m, 2H), 0.66 (t, 3H, *J* = 7.5 Hz). ^13^C-NMR (CDCl_3_, *δ*/ppm): 135.85, 122.79, 121.27, 48.63, 35.58, 31.07, 18.31, 12.28.

### 2. Cyclic Voltammetry Measurements

Measurements were performed in the temperature range 293–338 K. A typical three-electrode cell was employed, with the composition of a glassy carbon (GC) rod working electrode (0.1256 cm^2^), a platinum gauze counter electrode, and a silver/silver ion (0.1 mol·L^−1^ Ag^+^ in CH_3_CN) quasi-reference electrode. The electrochemical cell had a single leak-tight compartment and all the electrodes were placed in a compartment. The cell was kept under nitrogen atmosphere during entire study. Prior to measurements, the GC electrode were polished with a slurry aluminum oxide (0.05 µm), and were washed with deionized water and ethanol.

## Conclusions

The electrochemical behaviors of Eu(III), Sm(III), Dy(III) and Nd(III) in BmimBr at GC electrode from 293 K to 398 K were investigated. The cyclic voltammograms of Eu(III) exhibited quasi-reversible waves, while the cyclic voltammograms of Sm(III), Dy(III) and Nd(III) comprised of irreversible waves. An increase in current intensity was observed along with the increase of temperature. The diffusion coefficients, the transfer coefficients, and the activation energies of Ln(III) (Ln = Eu, Sm, Dy, Nd), and the charge transfer rate constants of Eu(III) were also calculated. The diffusion coefficient of Eu(III) is much larger than those of the other three lanthanide ions with the magnitude of ∼10^−8 ^cm^2^·s^−1^ at 293 K. Such a feature shows that there is a potential for selective separation of Eu from other lanthanides by electrochemical methods. The apparent standard potentials, *E* ^0^*_Eu(III)/Eu(II)_, and the standard Gibbs energy were also determined.
